# Quantum Nonlocality of Arbitrary Dimensional Bipartite States

**DOI:** 10.1038/srep13358

**Published:** 2015-08-25

**Authors:** Ming Li, Tinggui Zhang, Bobo Hua, Shao-Ming Fei, Xianqing Li-Jost

**Affiliations:** 1 College of the Science, China University of Petroleum, Qingdao 266580, P. R. China; 2 College of Mathematics and Statistics, Hainan Normal University, Haikou 571158, P. R. China; 3 School of Mathematical Sciences, LMNS, Fudan University, Shanghai 200433, P. R. China; 4 School of Mathematical Sciences, Capital Normal University, Beijing 100048, P. R. China; 5 Max-Planck-Institute for Mathematics in the Sciences, Leipzig 04103, Germany

## Abstract

We study the nonlocality of arbitrary dimensional bipartite quantum states. By computing the maximal violation of a set of multi-setting Bell inequalities, an analytical and computable lower bound has been derived for general two-qubit states. This bound gives the necessary condition that a two-qubit state admits no local hidden variable models. The lower bound is shown to be better than that from the CHSH inequality in judging the nonlocality of some quantum states. The results are generalized to the case of high dimensional quantum states, and a sufficient condition for detecting the non-locality has been presented.

Quantum mechanics is inherently nonlocal, as revealed by the violation of Bell inequality^1^. A bipartite quantum state may violates some Bell inequalities such that the local measurement outcomes can not be modeled by classical random distributions over probability spaces. Namely, the state admits no local hidden variable (LHV) model.

The nonlocality and quantum entanglement play important roles in our fundamental understandings of physical world as well as in various novel quantum informational tasks^2^,^3^. A quantum state without entanglement must admit LHV models^4^,^5^,^6^,^7^,^8^,^9^. However, not all the entangled quantum states are of nonlocality^10^,^11^,^12^,^14^. To show that a quantum state admits a LHV model, it is sufficient to construct such LHV model explicitly^10^,^12^. To show that a quantum state admits no LHV models, it is sufficient to show that it violates a Bell inequality^15^,^16^. Quantum states that violate Bell inequalities are also useful in building quantum protocols to decrease communication complexity^17^ and provide secure quantum communication^18^,^19^. Moreover, since the nonlocality is detected by the violation of Bell inequalities, quanum nonlocality could be quantified in terms of the maximal violation value for all Bell inequalities. However, it is a formidable task either to show that a state admits an LHV model, or to show that a state violates a Bell inequality.

Let *A*_*i*_ and *B*_*i*_, *i* = 1,2, …, *n*, be observables with respect to the two subsystems of a bipartite state, with eigenvalues ±1. Let *M* be a real matrix with entries *M*_*ij*_ such that 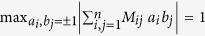. Denote 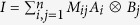 the corresponding Bell operator. Define


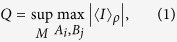


where 〈*I*〉_*ρ*_ = *tr*(*Iρ*) stands for the mean value of the Bell operator associated to state 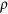. Obviously a quantum state *ρ* can never be described by a LHV model if and only if *Q* is strictly larger than 1.

In^10^,^11^,^12^,^13^,^14^, the authors have investigated the nonlocality of Werner states. For two-qubit Werner state 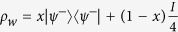, 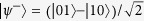, the quantity *Q* is proved to be 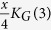 in^12^, where *K*_*G*_(3) is the Grothendieck’s constant of order three. However, since up to now one does not kown the exact value of the Grothendieck’s constant *K*_*G*_(3), *Q* is still is not known. The upper and lower bounds of the threshold value of this parameter *Q* have been refined by constructing better LHV models^10^,^11^,^12^ or by finding better Bell inequalities^13^,^14^.

In the paper we study the nonlocality of arbitrary two-qubit states and present an analytical and computable lower bound of the quantity *Q* by computing the maximal violation of a set of multi-setting Bell inequalities. The lower bound is shown to be better than that derived in terms of the CHSH inequality for some quantum states. We also present a sufficient condition that a high dimensional quantum state admits LHV models.

## Results

### Lower bound of *Q* for two-qubit quantum states

A two-qubit quantum state *ρ* can be always expressed in terms of Pauli matrices 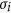, *i* = 1, 2, 3,


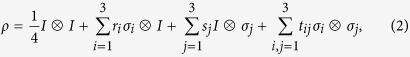


where 
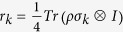
, 
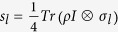
 and 
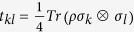
. We denote *T* the matrix with entries *t*_*ij*_.

The key point in computing *Q* is to find 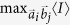 over all *M* under the condition 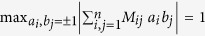. In^14^ a Bell operator has been introduced,


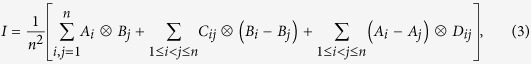


where *A*_*i*_, *B*_*j*_, *C*_*ij*_ and *D*_*ij*_ are observables of the form 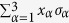 with 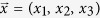 the unit vectors.

To find an analytical lower bound of 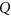, we consider infinite many measurements settings, *n* → ∞. Then the discrete summation in (3) is transformed into an integral of the spherical coordinate over the sphere *S*^2^ ⊂ *R*^3^. We denote the spherical coordinate of *S*^2^ by (*ϕ*_1_, *ϕ*_2_). A unit vector 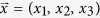 can parameterized by *x*_1_ = sin *ϕ*_1_ sin *ϕ*_2_, *x*_2_ = sin *ϕ*_1_ cos *ϕ*_2_, *x*_3_ = cos *ϕ*_1_. For any 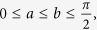 we denote 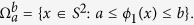


**Theorem 1:** For arbitrary two-qubit quantum state *ρ* given by (2), we have


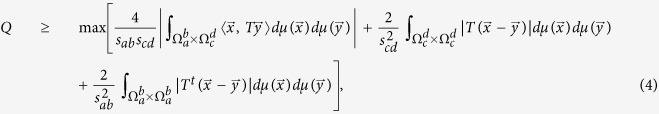


where *T*^*t*^ stands for the transposition of *T*, and 
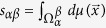
. The maximum on the right side of the inequality goes over all the integral area 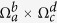 with 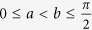 and 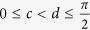.

See Methods for the proof of theorem 1.

The bound (4) can be calculated by parameterizing the integral in terms of the sphere coordinates. Once a two-qubit is given, the corresponding matrix *T* is given. And the bound is solely determined by *T*. This is similar to the CHSH inequality, where the maximal violation is given by the two larger singular values of *T*.

As an example, consider *T* = *diag*(*p*_1_, *p*_2_, *p*_3_), we have


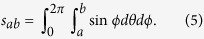



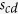
 in (4) are similarly given. The first two terms in 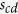 (4) are given by


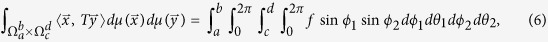



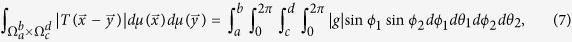


where


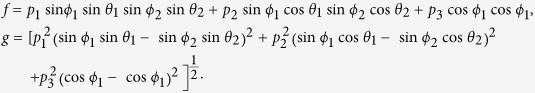


The last term in (4) is similarly to the second term, with *T* being replaced by *T*^*t*^.

**Figure 1:**
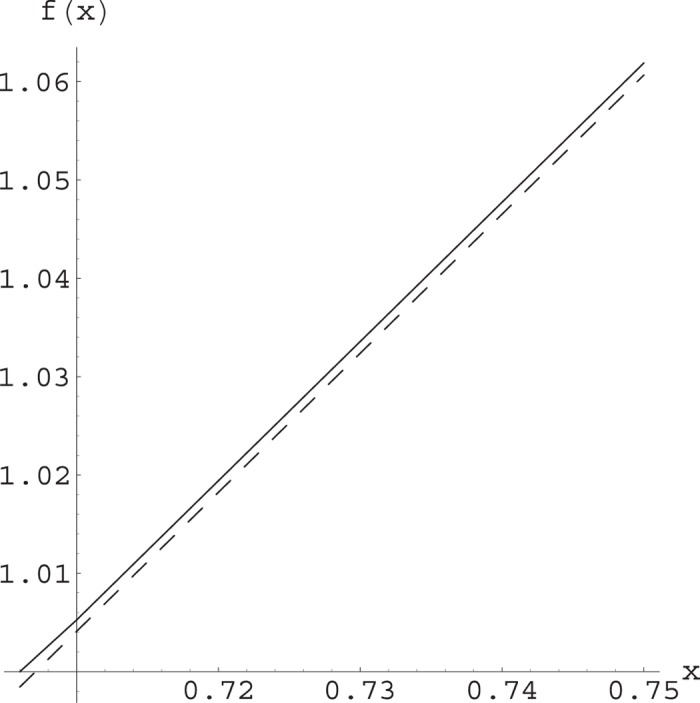
The lower bounds (denoted by *f*(*x*)) of *Q* in Theorem 1 (solid line) and that obtained from the CHSH inequality (dashed line).

Thus for any given two-qubit quantum state, by substituting *T* into the integral, we have the lower bound of *Q*. The maximum taken over 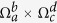 can be searched by varying the integral ranges. The Werner state considered in^10^,^11^,^12^,^13^,^14^ is a special case that *p*_1_ = *p*_2_ = *p*_3_ = *p*. From our Theorem 1, we have that for 0.7054 < *x* ≤ 1, the lower bound of *Q* is always larger than that is derived from the maximal violation of the CHSH inequality, see Fig. 1.

Let us now consider the generalized Bell diagonal two-qubit states in detail,


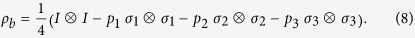


**Figure 2:**
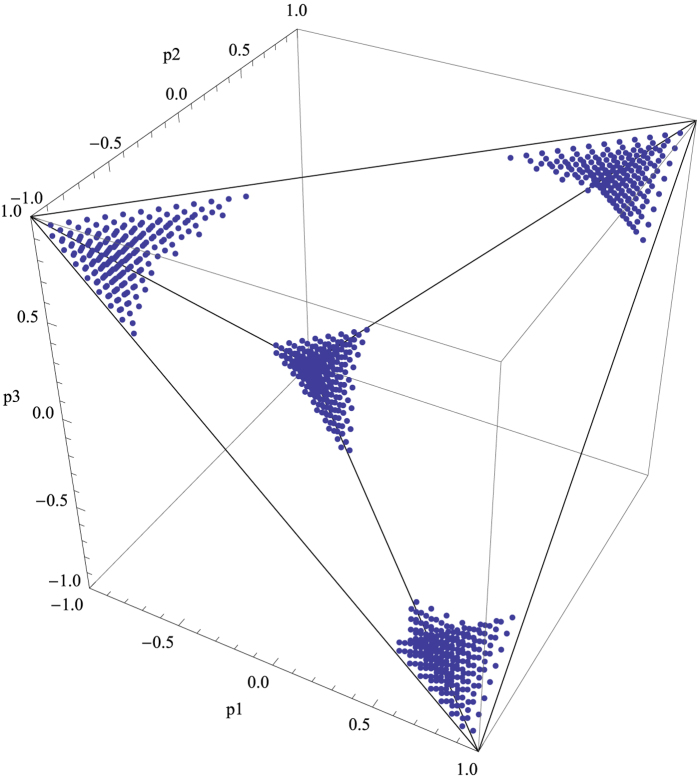
The quantum states 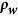 that admits no LHV models are listed by the points parameterized by (*p*_1_, *p*_2_, *p*_3_).

The positivity property requires that the parameters {*p*_1_, *p*_2_, *p*_3_} must be inside a regular tetrahedron with vertexes {−1, −1, 1}, {1, −1, −1}, {1, 1, 1}, {−1, 1, −1}. By computing the lower bound of *Q* according to Theorem 1, we detect the regions where the quantum states can never be described by LHV models, see Fig. 2.

**Figure 3:**
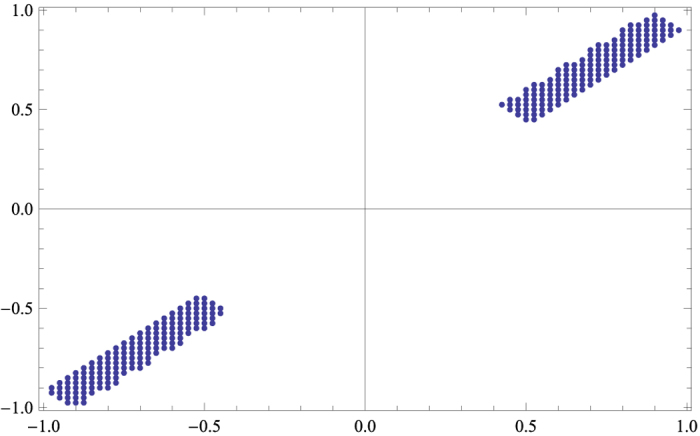
The same cross-sectional view of Fig. 2 for all p1 = 0.9, p2 = 0.9 and p3 = 0.9.

By setting *p*_1_ = 0.9, *p*_2_ = 0.9 and *p*_3_ = 0.9, one has the the cross-sectional view, see Fig. 3.

### High dimensional case

Generalizing our approach to high dimensional case, now we study the nonlocality of general *d* × *d* bipartite quantum states. To detect the nonlocality of a quantum state, the important thing is to find a ‘good’ Bell operator. For even *d*, we set Γ_1_, Γ_2_ and Γ_3_ to be block-diagonal matrices, with each block an ordinary Pauli matrix, 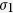, 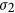 and 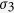 respectively, as described in^5^ for Γ_1_ and Γ_3_. When *d* is odd, we set the elements of the *k* th row and the 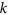
th column in Γ_1_, Γ_2_ and Γ_3_ to be zero, with the rest elements of Γ_1_, Γ_2_ and Γ_3_ being the block-diagonal matrices like the case of even *d*. Let Γ_0_ be a *d* × *d* matrix whose only nonvanishing entry is (Γ_0_)_*mm*_ = 1 for *m* ∈ 1, 2, …, *d*, for odd *d* and be a null matrix for even *d*. We define observables 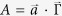 and 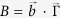, where 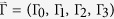, 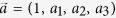 and 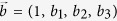 are vectors with norm 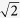. It is easy to check that the eigenvalues of the observables *A* and *B* are either 1 or −1.

We define the Bell operator to be


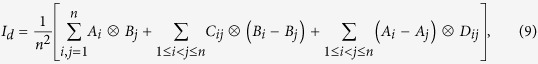


where *A*_*i*_, *B*_*j*_, *C*_*ij*_ and *D*_*ij*_ are observables of the form 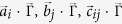 and 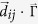 respectively; 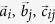 and 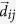 are vectors with norm 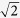.

The Bell operator (9) has the same structure as that in (3), but fits for *d*×*d* quantum system. For a *d* × *d* quantum state *ρ*, we set *γ* to be a matrix with elements *γ*_*ij*_ = *tr*(*ρ*Γ_*i*_ ⊗ Γ_*j*_), *i*, *j* = 0, 1, 2, 3. A lower bound of 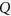 defined in (1) for *d*×*d* quantum system can be readily obtained as the follows.

**Theorem 2:** For any quantum state *ρ* in *d*×*d* quantum system 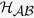, we have that


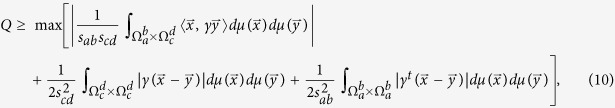


where *γ*^*t*^ stands for the transposition of *γ*, and 
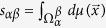
. The maximum on the right side of the inequality is taken over all the selection of integral area 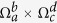 with 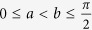 and 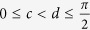.

See Methods for the proof of theorem 2.

According to the definition of 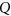 in (1), we have that if the lower bound for *Q* in theorem 2 is larger than one, then a quantum state in *d* × *d* bipartite quantum system can never be described by an LHV model. The bound can readily calculated, similar to the two-qubit case, once the matrix *γ* for state is given.

Let us consider the isotropic state *ρ*_*I*_^20^,^21^, a mixture of the singlet state 
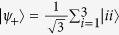
 and the white noise: 
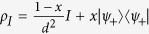
, 0 ≤ *x* ≤ 1. *ρ*_*I*_ is entangled for 
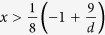
. For *d* = 3, *ρ*_*I*_ is entangled for *x* > 1/4. From Theorem 2, *ρ*_*I*_ is nonlocal for *x* > 0.7653.

**Figure 4:**
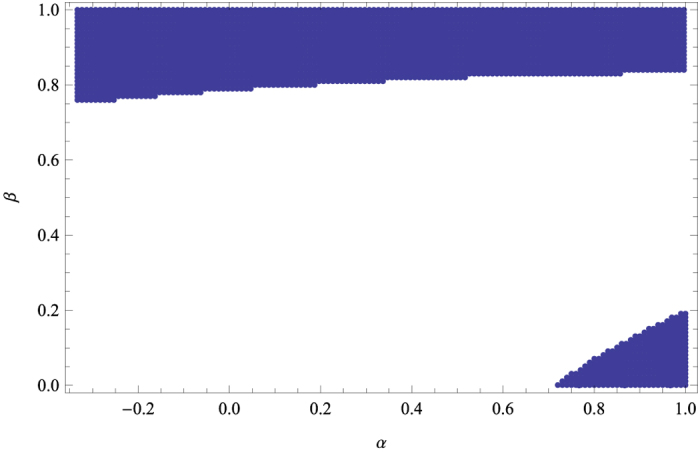
Quantum states 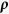 parameterized by 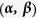 that admit no LHV model (blue regions).

As another example we consider the state *ρ* from mixing the singlet state 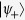 with 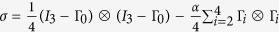, 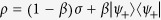. One can list by Theorem 2 the points that admit no LHV model, see Fig. 4.

## Discussions

Nowadays, quantum nonlocality is a fundamental subject in quantum information theory such as quantum cryptography, complexity theory, communication complexity, estimates for the dimension of the underlying Hilbert space, entangled games, etc.^22^. Thus it is a basic question to check and to qualify the nonlocality of a quantum state. We have derived an analytical and computable lower bound of the quantum violation by using a Bell inequality with infinitely many measurement settings. The bound is shown to be better than that is obtained from the CHSH inequality and the discrete models. Sufficient conditions for the LHV description of high dimensional quantum states have also derived. Apart from the computation of maximal violations for bipartite Bell inequalities, our methods can also contribute to the analysis of the nonlocality of multipartite quantum systems.

## Methods

### Proof of Theorem 1

For any two-qubit quantum state *ρ* given in (2), we have


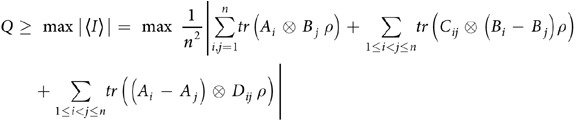



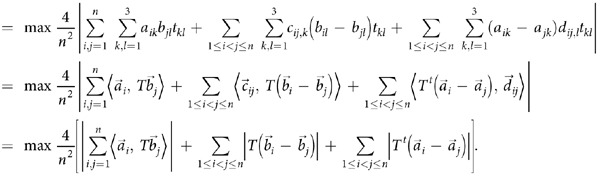


Under the limit *n* → ∞, we have


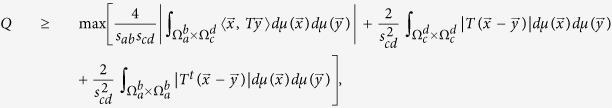


which proves (4).                     ■

### Proof of Theorem 2

With the special selected observables of the form 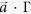 for 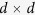 quantum systems, we have that


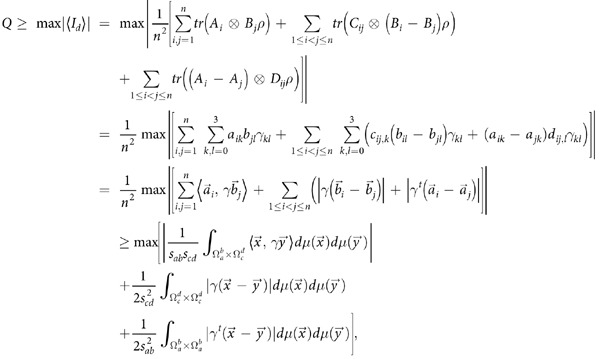


where in the last step, we have taken the limit *n* → ∞.   ■

## Additional Information

**How to cite this article**: Li, M. *et al*. Quantum Nonlocality of Arbitrary Dimensional Bipartite States. *Sci. Rep*. **5**, 13358; doi: 10.1038/srep13358 (2015).

## References

[b1] Bell J. S. On the Einstein Podolsky Rosen Paradox. Physics 1, 195–200 (1964).

[b2] Nielsen M. A. & Chuang I. L. Quantum Computation and Quantum Information (Cambridge University Press, Cambridge, England, 2000).

[b3] Di Vincenzo D. P. Quantum Computation. Science 270, 255–261 (1995).

[b4] Gisin N. Bell’s inequality holds for all non-product states. Phys. Lett. A 154, 201–202 (1991).

[b5] Gisin N. & Peres A. Maximal violation of Bells inequality for arbitrarily large spin. Phys. Lett. A 162, 15–17 (1992). 10.1103/physreva.46.4413 9908642

[b6] Popescu S. & Rohrlich D. Generic quantum nonlocality. Phys. Lett. A 166, 293–297 (1992).

[b7] Chen J. L., Wu C. F., Kwek L. C., & Oh C. H. Gisin’s Theorem for Three Qubits. Phys. Rev. Lett. 93, 140407 (2004). 15524776 10.1103/PhysRevLett.93.140407

[b8] Li M. & Fei S. M. Gisins Theorem for Arbitrary Dimensional Multipartite States. Phys. Rev. Lett. 104, 240502 (2010). 20867289 10.1103/PhysRevLett.104.240502

[b9] Yu S. X., Chen Q., Zhang C. J., Lai C. H. & Oh C. H. All entangled pure states violate a single Bell’s inequality. Phys. Rev. Lett. 109, 120402 (2012). 23005926 10.1103/PhysRevLett.109.120402

[b10] Werner R. F. Quantum states with Einstein-Podolsky-Rosen correlations admitting a hidden-variable model. Phys. Rev. A 40, 4277 (1989). 9902666 10.1103/physreva.40.4277

[b11] Barrett J. Nonsequential positive-operator-valued measurements on entangled mixed states do not always violate a Bell inequality. Phys. Rev. A 65, 042302 (2002).

[b12] Acin A., Gisin N., & Toner B. Grothendiecks constant and local models for noisy entangled quantum states. Phys. Rev. A 73, 062105 (2006).

[b13] Hua B. B., Zhou C. Q., Li M., Zhang T. G., Li-Jost X. Q. & Fei S. M. Towards Grothendieck constants and LHV models in quantum mechanics. J. Phys. A: Math. Theor. 48, 065302 (2015).

[b14] Vertesi T. More efficient Bell inequalities for Werner states. Phys. Rev. A 78, 032112 (2008).

[b15] Li M., Fei S. M. & LI-Jost X. Q. Bell Inequality, Separability and Entanglement Distillation. Chin. Sci. Bull. 56, 945–954 (2011).

[b16] Qi J. X. Zha, X. W. & Sun X. M. Testing the nonlocality of entangled states by a new Bell-like inequality. Sci. China-Phys. Mech. Astron. 56, 2236–2238 (2013).

[b17] Brukner C., Zukowski M. & Zeilinger A. Quantum Communication Complexity Protocol with Two Entangled Qutrits. Phys. Rev. Lett. 89, 197901 (2002). 12443149 10.1103/PhysRevLett.89.197901

[b18] Scarani V. & Gisin N. Quantum Communication between N Partners and Bell’s Inequalities. Phys. Rev. Lett. 87, 117901 (2001). 11531547 10.1103/PhysRevLett.87.117901

[b19] Acín A., Gisin N. & Scarani V. Security bounds in Quantum Cryptography using d-level systems. Quantum Inf. Comput. 3, 563–580 (2003).

[b20] Horodecki M. & Horodecki P. Reduction criterion of separability and limits for a class of distillation protocols. Phys. Rev. A 59, 4206 (1999).

[b21] Vollbrecht K. G. H. & Werner R. F. Entanglement measures under symmetry. Phys. Rev. A 64, 062307 (2001).

[b22] Junge M. & Palazuelos C. Large violation of Bell inequalities with low entanglement. Commun. Math. Phys. 306, 695–746 (2011).

